# Functional Ultrasound Imaging of Auditory Responses in Comatose Patients

**DOI:** 10.34133/research.0709

**Published:** 2025-05-15

**Authors:** Zihao Chen, Na Li, Caihua Xi, Jiaru He, Jiejun Zhu, Gang Wu, Jinzhao Xia, Chunlong Fei, Lei Sun, Hongzhi Xu, Zhihai Qiu

**Affiliations:** ^1^ Guangdong Institute of Intelligence Science and Technology, Hengqin, Zhuhai, Guangdong 519031, P. R. China.; ^2^ Department of Biomedical Engineering, The Hong Kong Polytechnic University, Hong Kong SAR 999077, P. R. China.; ^3^ Department of Neurosurgery, Huashan Hospital, Shanghai Medical College, Fudan University; National Center for Neurological Disorders; Shanghai Key Laboratory of Brain Function and Restoration and Neural Regeneration; Neurosurgical Institute of Fudan University; Shanghai Clinical Medical Center of Neurosurgery; Department of Neurosurgery & Neurocritical Care, Huashan Hospital, Shanghai Medical College, Fudan University, Shanghai, P. R. China.; ^4^ School of Microelectronics, Xidian University, Xi’an, Shannxi, P. R. China.

## Abstract

Bedside monitoring of brain function in severely brain-injured patients remains a critical clinical challenge. We demonstrate the translational potential of functional ultrasound (fUS) imaging for this purpose. In 6 comatose patients (Glasgow coma scale ≤ 8) with cranial windows after decompressive craniectomy, we used a 7.8-MHz transducer optimized for cortical depths of 1.5 to 4 cm to perform real-time fUS during auditory stimulation. We observed task-related increases in regional cerebral blood flow (rCBF) in relevant brain regions (*P* < 0.001, *t* test), which correlated with subsequent neurological recovery at 9-month follow-up. These findings establish fUS as a sensitive and portable tool for bedside brain function assessment, offering potential for improved prognostication, treatment guidance, and development of targeted rehabilitative strategies.

## Introduction

Decompressive craniectomy, a life-saving neurosurgical intervention for conditions such as traumatic brain injury [1] and stroke [2], reduces intracranial pressure [3] and prevents brain herniation [4]. While many patients experience gradual neurological recovery after decompressive craniectomy, a substantial subset suffers prolonged disorders of consciousness (DOC), including coma and persistent vegetative state [5]. This poses an important challenge for clinicians, as accurate prognostication and individualized treatment planning are critically dependent on precise assessment of residual brain function [6–10].

Precise assessment of residual brain function in these patients is crucial for guiding clinical decisions, optimizing recovery, and informing rehabilitation strategies. However, current bedside assessment methods, such as the Glasgow coma scale (GCS) [11] and Coma Recovery Scale-Revised (CRS-R) [12], rely on subjective behavioral observations, limiting their sensitivity to subtle neurophysiological activity and covert consciousness [13,14]. Objective neuroimaging techniques like electroencephalography (EEG) [15], functional magnetic resonance imaging (fMRI) [16], and positron emission tomography–computed tomography (PET-CT) [17] offer greater detail, but their high cost, limited portability, invasiveness (e.g., radiation exposure with PET-CT), and inability to provide continuous, high-resolution monitoring at the bedside restrict their widespread clinical utility in this vulnerable population, who often require prolonged intensive care [18,19]. Therefore, a portable, sensitive, and practical bedside neuroimaging technique capable of long-term monitoring in this unique clinical context is urgently needed. This highlights a critical unmet need for a brain function monitoring tool specifically tailored to the challenges presented by post-decompressive craniectomy patients.

Functional ultrasound (fUS) imaging [20] offers a promising solution. This emerging neuroimaging modality combines high spatiotemporal resolution (up to 10 Hz, ~100 μm) with portability and cost-effectiveness, enabling real-time assessment of brain activity through neurovascular coupling [21]. By measuring changes in cerebral blood volume (CBV) reflecting neuronal activity, fUS can detect subtle signals even in deep brain regions and small neural populations. Its successful application across species, from rodents to nonhuman primates and humans neonates, shows superior sensitivity to functional changes [22–29]. Demonstrated single-trial sensitivity in nonhuman primates suggests that fUS is well-suited for monitoring residual brain activity in comatose patients, highlighting its translational potential [30]. While fUS application in intact skulls is limited by skull-induced acoustic impedance, post-decompressive craniectomy patients with cranial windows provide an ideal opportunity to leverage fUS's capabilities*.* These cranial windows offer direct acoustic access to the brain, enabling high-quality fUS imaging of functional activity in this critical clinical population [26]. What is more, as the basis for fUS, bedside Doppler imaging in comatose patients has also recently been reported, which further proves the feasibility of fUS [31].

The limitations of fUS for assessing consciousness underscore the clinical need for novel approaches like fUS. Behavioral assessments (e.g., GCS and CRS-R), while essential, are subjective and insensitive to subtle changes in consciousness, particularly in minimally conscious states [32]. Neuroimaging techniques like CT and MRI, while informative, are often costly, inaccessible at the bedside, and unsuitable for continuous monitoring [33]. EEG, although portable, can be challenging to implement in patients with head injuries due to the need for secure electrode placement [34]. fUS offers a compelling alternative by providing a portable, noninvasive, and relatively inexpensive method for real-time monitoring of brain function at the bedside.

Here, we investigate the feasibility of bedside real-time fUS to detect subtle brain activity in patients following decompressive craniectomy. We optimized fUS parameters, including transducer frequency (7.8 MHz), to target the left superior temporal gyrus (STG) (1.5 to 4 cm depth) during auditory stimulation. In 3 severely comatose patients (GCS ≤ 8), through real-time implementation to obtain fUS imaging, we demonstrate task-induced increases in regional CBV in expected functional areas, establishing fUS as a potentially robust and clinically translatable tool for bedside real-time brain function monitoring and offering a foundation for future studies in consciousness assessment and neurorehabilitation.

## Results

### fUS imaging capabilities

**Fig. 1.**
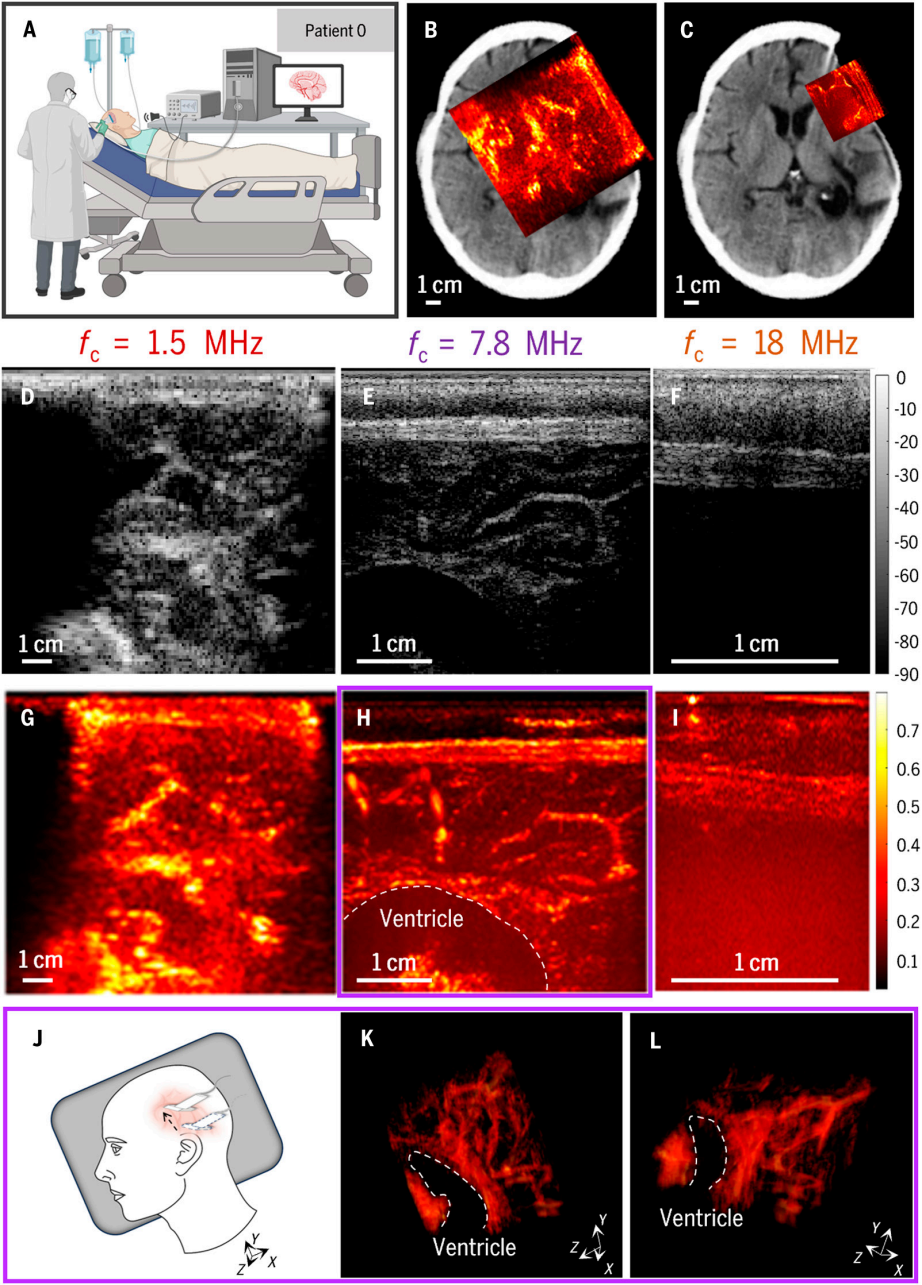
Bedside fUS imaging in a patient with a cranial window. (A) Representative photograph of fUS signal acquisition in a patient with a cranial window on patient 0. (B and C) Co-registration of the fUS imaging plane (red square) with an anatomical CT image for patient 0. Power Doppler images acquired through transducers with 1.5-MHz (B) and 7.8-MHz (C) center frequencies. Scale bar, 1 cm. (D to F) Compound ultrasound plane images acquired using transducers with different center frequencies: 1.5 MHz (D), 7.8 MHz (E), and 18 MHz (F). Scale bar, 1 cm. (G to I) fUS images acquired with 1.5-MHz (G), 7.8-MHz (H), and 18-MHz (I) transducers. Dashed line highlights the ventricle. Scale bar, 1 cm. (J) Schematic diagram of 4D fUS imaging scan on patient 0. (K and L) Different angular views of 3D fUS imaging using a 7.8-MHz transducer. Direction *X*: Linear element direction of the ultrasound transducer. Direction *Y*: Moving direction of the ultrasound transducer. Direction *Z*: Direction perpendicular to the ultrasound transducer. The dashed line highlights the ventricle. Scale bar, 1 cm.

Bedside fUS imaging (Fig. 1A) demonstrated its versatility in capturing brain activity at various depths and spatial resolutions by adjusting the center frequency on patient 0 (Fig. 1B and C). Lower frequency like 1.5 MHz offered deeper penetration (up to 10 cm; Fig. 1B) suitable for visualizing larger brain regions, including cortical surfaces, central areas, and parts of deeper structures on patient 0 (Fig. 1D and G). However, the spatial resolution at this frequency was lower (850 μm), allowing visualization only of major arteries and veins exceeding 1 mm in diameter.

Increasing the frequency to 7.8 MHz enhanced spatial resolution to 300 μm (Fig. 1E and H), enabling clear visualization of medium-sized vessels like tertiary branch arteries, small veins, and larger microvessels (~300 μm) on patient 0. This frequency also provided a general representation of the capillary network distribution (Fig. 1C). However, the imaging depth decreased to 4 cm compared to the lower frequency.

Further increasing the frequency to 18 MHz offered the highest spatial resolution (200 μm; Fig. 1F and I) on patient 0, potentially valuable for detailed anatomical studies. However, limitations arose due to significant attenuation by the scalp and dura mater in adult brains, hindering direct brain imaging with noninvasive transcranial methods at this frequency.

By adjusting the transducer position (Fig. 1J), fUS can be used to acquire 3-dimensional (3D) cerebral blood flow images through the cranial window. Figure 1K and L showcases different perspectives of 3D blood flow images obtained in patient 0 using a 7.8-MHz transducer. These images clearly depict the ventricles and the distribution of blood flow within the accessible brain region.

The contrast-to-noise ratio (CNR) of the image exceeds 20 dB, meeting the requirements for Doppler imaging. The minimum full width at half maximum (FWHM) achieved is 360 μm (Fig. S1).

### Auditory-evoked responses in the STG of severely comatose patients

**Fig. 2.**
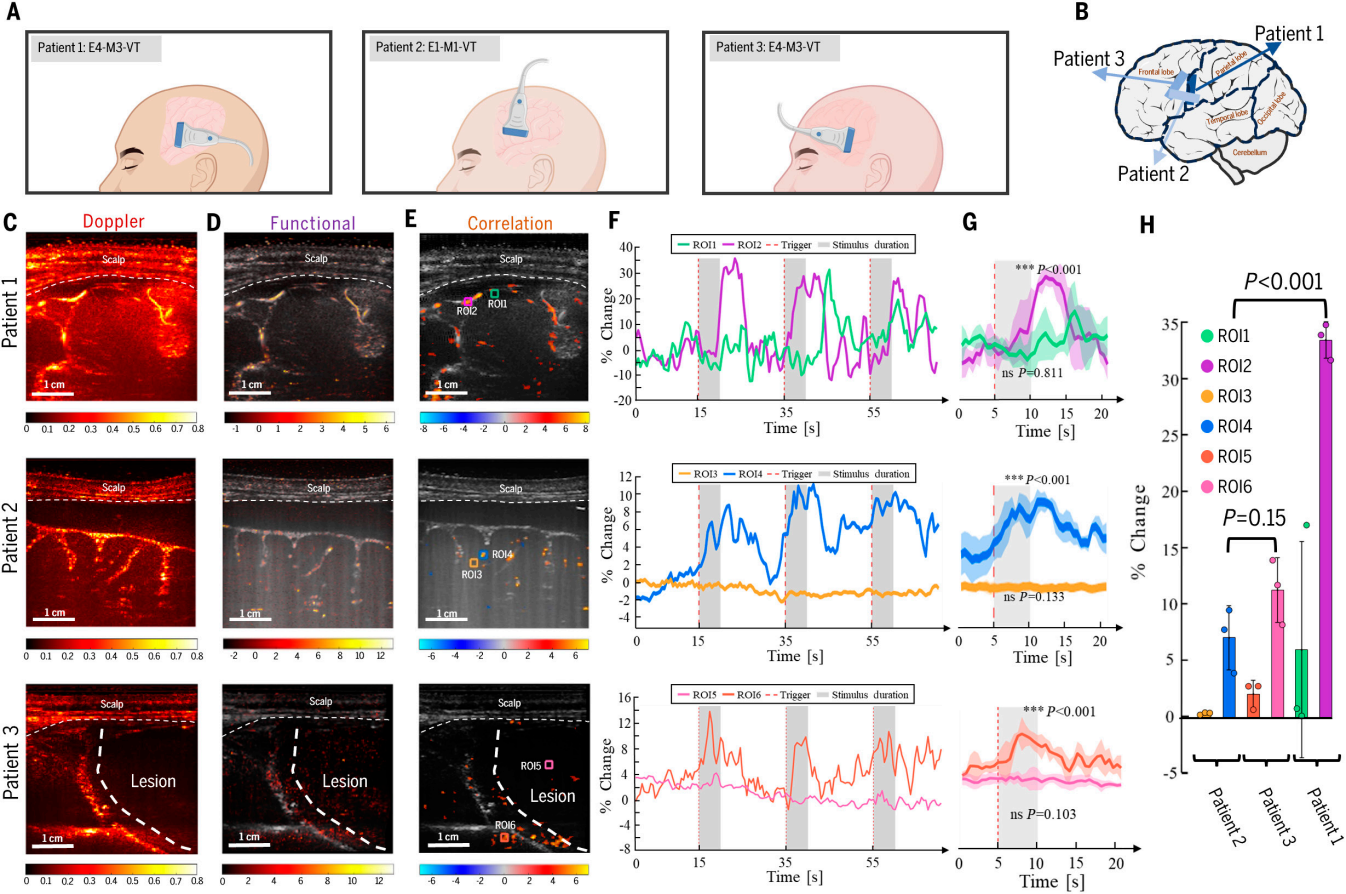
fUS imaging reveals auditory-evoked brain activity in STG of unconscious patients. (A) Schematic diagram of transducer placement during fUS signal acquisition for patients 1 to 3. (B) Schematic diagrams illustrating the STG we are interested and the position of the ultrasound transducer on the brain for patients 1 to 3. (C) Power Doppler images of the vascular anatomy within the imaging plane for patients 1 to 3. Dashed lines indicate the scalp and lesion region. Scale bar, 1 cm. (D) fUS images depicting changes in CBV within the imaging plane for patients 1 to 3. Dashed lines indicate the scalp. Scale bar, 1 cm. (E) fUS images highlighting brain regions activated by auditory stimuli. Dashed lines indicate the scalp. The color scale represents the T-score statistical parametric map, with voxels colored if *P* < 0.001. Colored boxes indicate ROIs: ROI1, ROI3, and ROI5 are nonreactive regions, and ROI2, ROI4, and ROI6 are reactive regions. Scale bar, 1 cm. (F) Time courses of CBV changes within the defined ROIs for patients 1 to 3. Red dashed lines indicate the onset of auditory stimuli. Gray shaded areas highlight the stimulation duration. (G) Bar graphs showing the mean CBV changes during stimulation periods for patients 1 to 3. Error bars represent standard deviation. The *t* test was conducted to compare CBV changes before and after stimulation. CBVs in ROI1, ROI3, and ROI5 showed no significant differences, while ROI2, ROI4, and ROI6 demonstrated significant increases during auditory stimulation (*P* < 0.001). (H) Bar graph comparing CBV changes between patients 1, 2, and 3.

Three craniectomized comatose patients (patients 1 to 3) with cranial windows positioned over the STG were selected for fUS imaging during auditory stimulation. The STG is a critical brain region involved in auditory processing, language comprehension, and social perception, playing a crucial role in self-awareness [35]. Patients 1 to 3 were presented with severe coma, as indicated by GCS scores of E4-M3-VT, E1-M1-VT, and E4-M3-VT, respectively (Fig. 2A). Given STG’s proximity to the brain surface (typically 2 to 5 mm depth), a 7.8-MHz transducer, offering a 4-cm penetration depth, 300-μm spatial resolution, and 2-Hz temporal resolution, was chosen for optimal imaging. Figure 2B illustrates the transducer placement relative to the brain.

During the presentation of three 5-s auditory stimuli separated by 20-s intervals, patients were monitored in a free-field environment. Resting-state cerebral blood flow images for patients 1 to 3 are shown in Fig. 2C. Normalized Doppler data were used to generate brain perfusion change maps, identifying functionally active voxels (Fig. 2D). Statistical analysis using a general linear model (GLM) and Student’s *t* test identified regions exhibiting significant task-related functional activation (Fig. 2E).

Time-series analysis of regional cerebral blood flow (rCBF) in response to auditory stimulation (Fig. 2F) revealed distinct responses in predefined regions of interest (ROIs; marked in Fig. 2E). Each ROI was fixed in size at approximately 2 mm × 2 mm. Reactive and nonreactive ROIs were selected from regions within the fUS images displaying well-defined vasculature, corresponding to areas with the highest and lowest correlation values, respectively. As expected, nonreactive ROIs (ROI1, ROI3, and ROI5) showed no significant changes in rCBF during stimulation (ROI1: mean difference = 0.235%, *P* = 0.811; ROI3: 0.064%, *P* = 0.133; ROI5: 0.30%, *P* = 0.103). In contrast, reactive ROIs (ROI2, ROI4, and ROI6) exhibited significant increases in rCBF in response to auditory stimulation (ROI2: 11.95%, *P* < 0.001; ROI4: 3.69%, *P* < 0.001; ROI6: 3.19%, *P* < 0.001). Auditory stimulation evoked distinct changes in rCBF within the predefined ROI during each of the 3 stimulation periods (Fig. 2G). Quantification of these rCBF changes (Fig. 2H) revealed a clear correlation between response intensity and clinical assessment.

### Auditory did not evoke obvious responses in the superior frontal gyrus of severely comatose patients

**Fig. 3.**
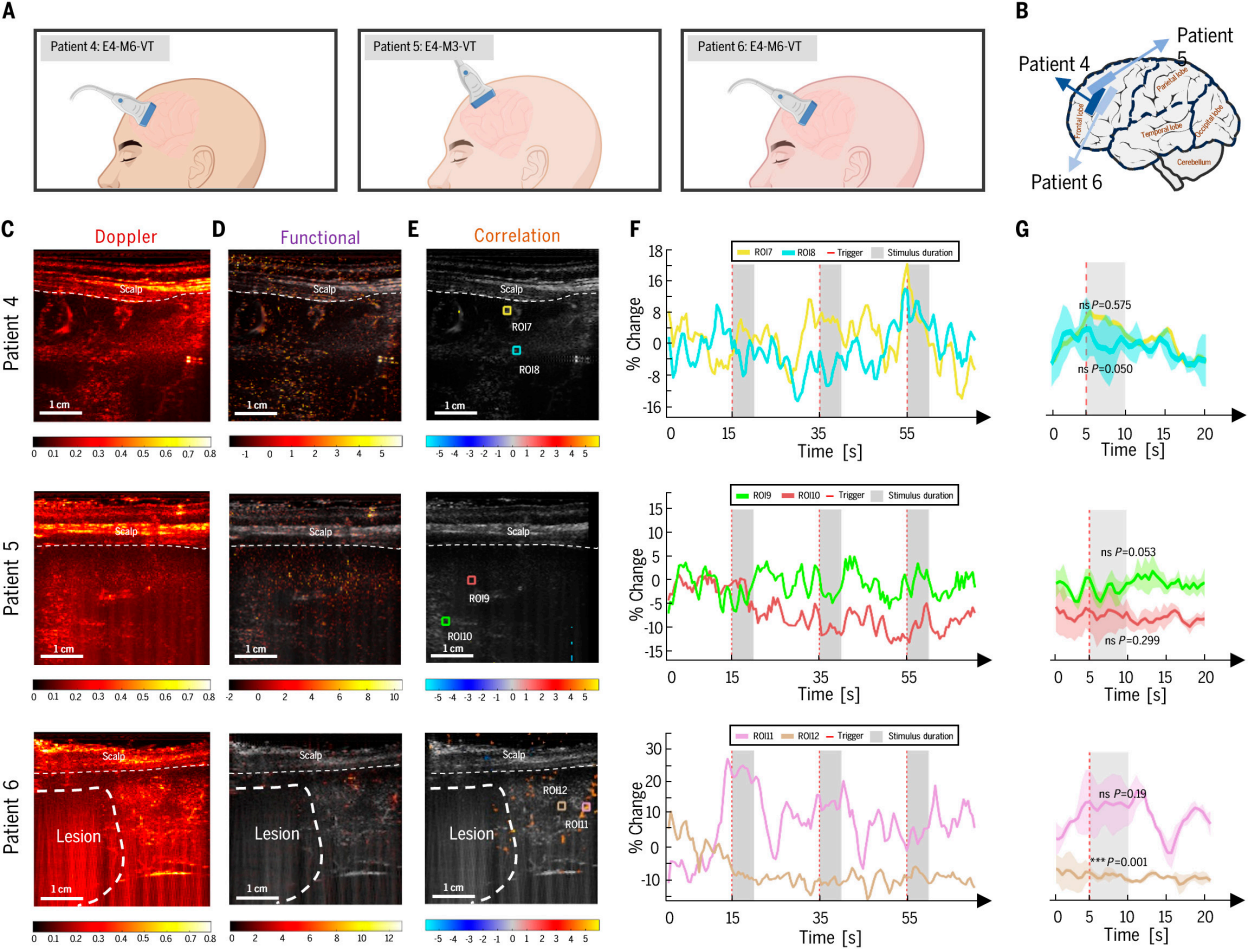
fUS imaging reveals no auditory-evoked brain activity in the SFG of an unconscious patient. (A) Schematic diagram of transducer placement during fUS signal acquisition for patients 4, 5, and 6. (B) Schematic illustration of the ultrasound transducer position on the brain, demonstrating coverage of the SFG. (C) Power Doppler image of the vascular anatomy within the imaging plane. Dashed line indicates the scalp. Scale bar, 1 cm. (D) fUS image depicting changes in CBV within the imaging plane. Dashed line indicates the scalp. Scale bar, 1 cm. (E) fUS image highlighting brain regions activated by auditory stimuli. Dashed line indicates the scalp. The color scale represents the T-score statistical parametric map, with voxels colored if *P* < 0.001. Colored boxes indicate ROIs: ROI7, ROI8, ROI9, ROI10, ROI11, and ROI12. Scale bar, 1 cm. (F) Time course of CBV changes within the defined ROIs. Red dashed lines indicate the onset of auditory stimuli. Gray shaded areas highlight the stimulation duration. (G) Bar graph showing the mean CBV changes during stimulation periods. Error bars represent standard deviation. The *t* test revealed no significant differences in CBV changes between ROI7 and ROI8, ROI9 and ROI10, and ROI11 and ROI12 during auditory stimulation.

To further validate these findings, other 3 patients (patients 4, 5, and 6) with a cranial window over the superior frontal gyrus (SFG) were examined. The SFG plays a crucial role in higher-order cognitive functions, including self-awareness, motor planning, and emotional regulation [35]. Patient 4 presented with a GCS score of E4-M6-VT, indicating a state of moderate coma. fUS imaging was performed as described above, revealing no significant brain activity in the SFG region in response to auditory stimuli (Fig. 3C to G, the line of patient 4). Similar results were shown in patients 5 and 6 (Fig. 3C to G, the line of patients 5 and 6). The analysis revealed no significant changes in blood flow within 2 selected ROIs (ROI7 and ROI8, ROI9 and ROI10, and ROI11 and ROI12) during auditory stimulation (ROI7: 0.781%, *P* = 0.575; ROI8: −2.23%, *P* = 0.050; ROI9: 0.649%, *P* = 0.526; ROI10: −7.27%, *P* = 0.299; ROI10: 0.649%, *P* = 0.526; ROI11: −7.27%, *P* = 0.299).

## Discussion

This study demonstrates the feasibility of bedside fUS imaging for detecting subtle brain functional activity in severely comatose, post-craniectomy patients. By comparing ultrasound transducers with varying center frequencies, we optimized imaging parameters for penetration depth and spatial resolution through the cranial window. This optimization was crucial for guiding transducer selection and demonstrating the feasibility of noninvasive brain function imaging in this challenging clinical setting. Using a 7.8-MHz transducer, we achieved a temporal resolution of 2 Hz and a spatial resolution of 300 μm, successfully recording auditory-evoked responses in 3 comatose patients (GCS ≤ 8) in STG.

To further strengthen our conclusions, additional fUS data were acquired from 3 patients with recordings in SFG. In particular, patient 6, although approaching recovery of consciousness—as indicated by spontaneous eye opening, eye movement, and observable responses to external commands—did not show auditory-related functional responses in the SFG during the experiment. Notably, fUS detected spontaneous neural activity in the SFG region; however, this activity was not temporally correlated with the auditory stimulation protocol (Fig. 3E, patient 6). These findings support the notion that in patients with moderate coma, higher-order cognitive functions, such as those mediated by the SFG, may be impaired, limiting their capacity to engage in complex auditory processing [35]. These demonstrate the superior sensitivity of fUS for detecting task-induced responses and highlight its potential for both research and clinical applications, even in patients with partial brain damage in specific regions (patient 3).

Our findings are consistent with previous studies utilizing fUS in neurosurgical contexts. Rabut et al. [26] and Soloukey et al. [36] demonstrated real-time fUS monitoring of brain activity in patients with sonolucent skull implants during gaming and walking, respectively. These studies, along with preclinical work in primates [37], underscore the excellent spatiotemporal resolution and sensitivity of fUS to task-related brain states. Our results extend these findings by demonstrating the feasibility of fUS in the acute post-craniectomy setting, even in the absence of a specialized implant. This is a crucial distinction as it expands the potential applicability of fUS to a broader patient population.

While higher frequency transducers offer the potential for increased spatial resolution, our attempts with an 18-MHz transducer were unsuccessful in visualizing clear cortical signals through the cranial window. This observation aligns with findings by Dizeux et al. [37], who achieved high-resolution cortical signal decoding in primates using a 15-MHz transducer through a cranial window. This comparison highlights the significant attenuation of high-frequency ultrasound by the dura mater and scalp, a critical consideration for future fUS studies in nonimplanted settings. Further research is needed to optimize high-frequency fUS for transcranial applications [38].

Our study demonstrates the potential of fUS to address a critical unmet clinical need: objective and continuous monitoring of brain function in comatose patients. This has profound implications for improving diagnostic accuracy, informing prognosis, and guiding personalized treatment strategies. We envision fUS playing a crucial role across the continuum of care, from intraoperative monitoring to post-acute rehabilitation. Future studies should investigate the integration of fUS with neuromodulation techniques for closed-loop interventions, potentially leading to more effective rehabilitation strategies for patients with DOC.

## Materials and Methods

### Study design

Assessing the level of consciousness in patients with severe coma following decompressive craniectomy is a critical clinical challenge. This study aimed to evaluate the feasibility of using fUS to assess and monitor consciousness in patients with severe coma caused by conditions such as traumatic brain injury. For the human study, all procedures were approved by the Institutional Review Boards of Huashan Hospital. Four patients who had undergone decompressive craniectomy were recruited for the study, and they signed a written informed consent form to participate in the study. First, we assessed the performance of different ultrasound transducers for imaging the brain through the cranial window created by decompressive craniectomy. This step was essential for selecting the most suitable ultrasound frequency to ensure robust and reliable data collection. Additionally, we developed a robust testing system to demonstrate that fUS technology can effectively monitor brain function recovery in the STG of severely comatose patients, offering valuable supplementary insights for the assessment of minimal consciousness.

### fUS imaging

fUS leverages neurovascular coupling, which links neuronal activity to changes in cerebral blood flow [39]. By acquiring power Doppler images that reflect blood flow distribution, fUS detects changes in brain activity [33]. We used 3 different ultrasound transducers: a custom-built 1.5-MHz transducer (128 elements, 850-μm pitch), a commercial 7.8-MHz transducer (L11-5v, 128 elements, Verasonics Inc.), and an 18-MHz transducer (L22-14vX, 128 elements, Verasonics Inc.). The maximum imaging depth decreases with the increase of frequency, and the depths were 100, 40, and 20 mm. fUS was performed on a Verasonics platform controlled by a modified MATLAB-based Miniscan interface [22]. Each power Doppler image block was generated by processing 250 compounded B-mode image frames with a frame rate of 500 Hz. To enhance the signal-to-noise ratio (SNR), each frame underwent triple temporal averaging, and compounding was conducted using 7 angles (−6°, −4°, −2°, 0°, 2°, 4°, and 6°). The pulse repetition frequency was approximately 10 kHz. Singular value decomposition (SVD) filtering was applied to remove stationary tissue artifacts from each block, isolating the blood flow signals to produce power Doppler images. Based on a comparative evaluation of images acquired from patient 0 under varying SVD threshold settings, a threshold of 13% was selected and subsequently applied across all experiments [26,31]. The final fUS imaging frame rate was 2 Hz, with images displayed in real-time. In addition, a sequential plane-scanning strategy was utilized to acquire 4D fUS data, with each imaging plane collected under consistent stimulation paradigms to ensure comparability across volumes (Fig. 1J to L).

### fUS data processing

Data processing was adapted from previously established clinical fUS methods [26], using a GLM to extract voxels responsive to auditory stimulation. Among the transducers, data collected with the 7.8-MHz transducer (L11-5v) were the most suitable. Since the patients lacked cranial bones, nonrigid motion artifacts from the scalp and surrounding tissues were corrected during preprocessing [40]. Each fUS image was spatially smoothed using a 2D Gaussian filter (σ = 1), and voxel signals were normalized to eliminate mean values [26,41]. Temporal smoothing was achieved with a moving average filter spanning 5 time points. Auditory-evoked responses were modeled using a gamma hemodynamic response function (HRF) with parameters τ = 0.7 s, δ = 1.5 s, and *n* = 3 [26,42]. The HRF was used as a regressor in the GLM for each normalized voxel, and statistical significance was determined at a threshold of *P* < 10^−3^, with corrections applied using the false discovery rate (FDR). For selected ROIs, multiple auditory-evoked responses were analyzed using a *t* test to compare resting and task states, with a significance threshold set at *P* < 10^−5^. Within each ROI, spatial averaging was first applied to the voxel data in the fUS image sequence. Baseline activity was defined using the prestimulus period, and functional responses over time were computed by calculating the standard *z* score, yielding time-resolved activation response.

### Human participants

We recruited 7 patients with severe coma who had undergone decompressive craniectomy at Shanghai Huashan Hospital. All participants’ family members provided informed consent for this study, which involved recording fUS signals during auditory stimulation. The study design and procedures were approved by the Institutional Review Boards of the Guangdong Institute of Intelligence Science and Technology and the National Neurological Diseases Research Center at Huashan Hospital. All fUS data acquisition was conducted at Huashan Hospital under clinical supervision.

### Auditory stimulation

CT images were used to identify the approximate brain regions beneath the cranial window. After sterilizing the transducer and the patient’s scalp, a neurosurgeon positioned the transducer on the exposed brain region. The position was fine-tuned based on real-time power Doppler images. The auditory stimulation consisted of three 5-s auditory stimulation (wide-band sound clicks, ~70 dBL) interspersed with 20-s rest intervals, preceded by a 20-s baseline rest period. After the test, the patient’s scalp was re-sterilized.

### Statistical analysis

All raw data are provided in the Supplementary Materials. Unless otherwise specified, statistical significance was defined as *P* < 0.001. Comparisons between groups were performed using 2-sided Student’s *t* tests. All statistical analyses were conducted using MATLAB 2024a. For GLM analyses, *P* values were corrected for multiple comparisons using the FDR method.

## Data Availability

All data associated with this study are available in the paper or the Supplementary Materials. Code used to collect fUS data, analyze fUS time series, and generate the key figures and results is publicly available on GitHub at https://github.com/Alex-czh/brain_fus, and an archived version is stored on Zenodo at https://doi.org/10.5281/zenodo.14511961 [43].
