# Supplementary figures and images for: Functional Ultrasound Imaging of Auditory Responses in Comatose Patients
Source: Research (Wash D C). 2025 May 15;8:0709. doi: 10.34133/research.0709 (PMC12079190; doi:10.34133/research.0709)

**Supplementary**


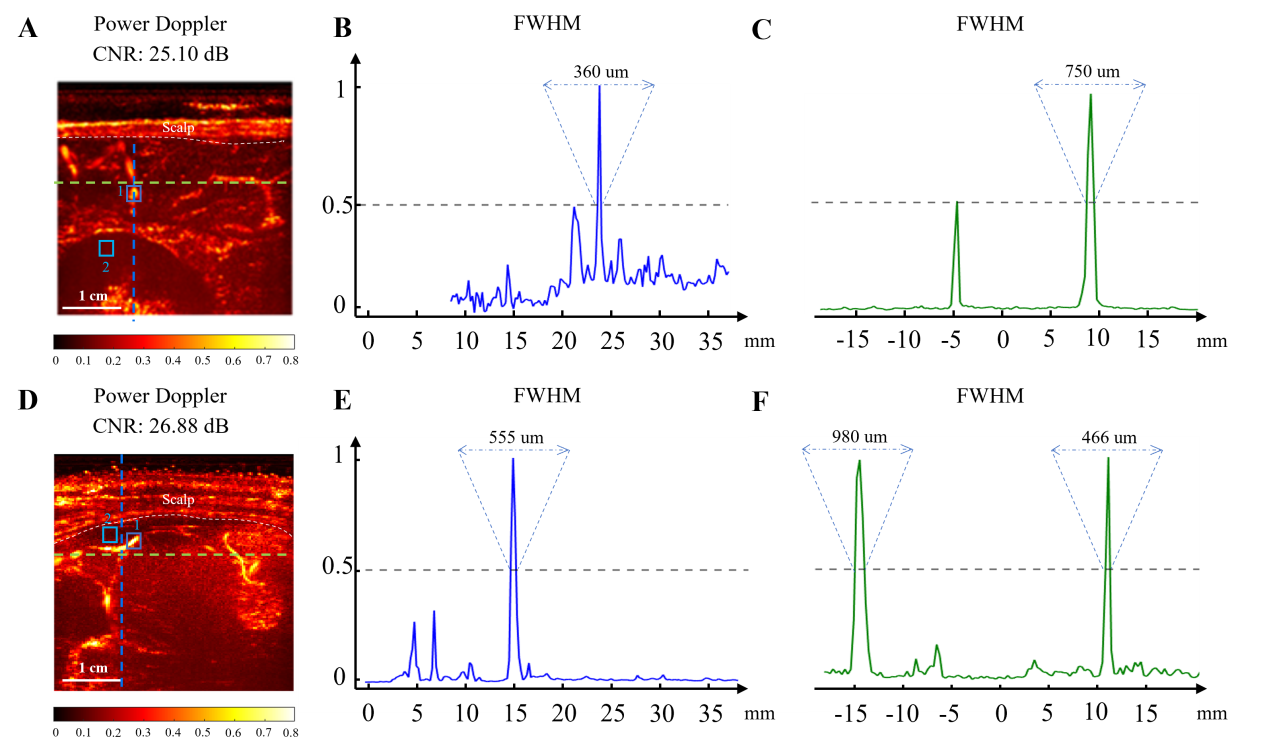


**Figure 1** Quantitative analysis for fUS.

Among them, CNR were calculated by:

Supplement: Supplementary 1 — Fig. S1 [file research.0709.f1.docx]
